# YAP1 inhibition protects retinal vascular endothelial cells under high glucose by inhibiting autophagy

**DOI:** 10.1515/biol-2022-0970

**Published:** 2025-12-24

**Authors:** Yu-Shan Li, Wen-Qiang Liu, Hong-Dan Yu, Zhong-Fu Zuo, Xue-Zheng Liu

**Affiliations:** Department of Anatomy, Histology and Embryology, Jinzhou Medical University, Jinzhou, 121001, China; Liaoning Key Laboratory of Diabetic Cognitive and Perceptive Dysfunction, Jinzhou Medical University, 121001, Jinzhou, China

**Keywords:** endothelial cell injury, YAP1, diabetic retinopathy

## Abstract

Diabetic retinopathy (DR) is a frequent diabetes‐associated microvascular disorder, and its underlying mechanism of pathogenesis remains unclear. Previous studies have suggested that YAP1 is involved in endothelial activation and vascular inflammation. In the present study, we explored the biological role of YAP1 in retinal vascular endothelial cells (RVECs) in response to high glucose (HG). YAP1 expression was first evaluated in retinal endothelial cells cultured with HG. In retinal endothelial cells, the consequences of HG exposure and the alteration of YAP1 expression were systematically evaluated, focusing on cell growth, survival, tube formation, migration, and activation of autophagy. To further evaluate the protective effect of YAP1 inhibition on retinal endothelial cells *in vivo*, a streptozocin-induced DR mouse model was employed. First, we discovered that YAP1 expression was dose-dependently elevated under glucose treatment in retinal endothelial RF/6A and human primary retinal endothelial cells. Accordingly, suppression of YAP1 exhibited a protective role in HG-treated retinal endothelial cells by decreasing cell viability and suppressing apoptotic deaths. Moreover, inhibition of YAP1 impairs migration and tube formation of retinal cells and alleviates autophagy in response to HG. Additionally, YAP1 knockdown reversed glucose-induced nucleus translocation of NF-κB. This study demonstrated that inhibition of YAP1 exhibits a protective role against HG-induced RVEC injury, representing a novel target for the management and therapy of DR in clinics.

## 1 Introduction

Characterized predominantly by hyperglycemia, diabetes mellitus (DM) constitutes a metabolic syndrome and is listed as the third chronic disease in prevalence, following only neoplasms and cerebrovascular and cardiovascular diseases [1]. As one of the foremost vascular complications arising from DM, diabetic retinopathy (DR) is a major contributor to visual impairment in the adult population. The onset of vascular complications in diabetes is intricately linked to endothelial dysfunction, which is primarily induced by high glucose (HG) [2,3]. It is reasonable to elucidate the mechanism by manipulating the vascular endothelial cells and identifying novel targets for DR management and treatment in clinics.

Autophagy represents a coordinated lysosomal degradation mechanism, activated in response to diverse stimuli, crucial for maintaining cellular homeostasis [4]. There is a compelling corpus of evidence that demonstrates a tight interconnection between autophagy activation in retinal vascular endothelial cells (RVECs) and the angiogenic processes in the retina, pivotal in the development of DR [5,6]. Therefore, unraveling the molecular intricacies of the autophagic process within the context of DR pathophysiology is of paramount importance.

YAP1 (Yes-Associated Protein 1) is a transcriptional regulator that is involved in cell proliferation, apoptosis, and autophagy, thereby controlling tissue regeneration and organ development [7]. When the Hippo signaling pathway is active, it results in the ubiquitin-mediated degradation of YAP1 in the cytoplasm. On the other hand, the suppression of this pathway leads to YAP1 activation and its nuclear translocation, which in turn triggers the expression of specific target genes. Emerging studies have elucidated the significant involvement of YAP1 in controlling endothelial activation and vascular inflammation, central to the pathogenesis of cardiovascular diseases [8,9]. Nonetheless, the potential implication of YAP1 in RVECs in the context of HG exposure has not yet been established.

Given this, the present study evaluated YAP1 expression in RVECs under HG. In addition, the biological effects of YAP1 on cell vitality, apoptosis, migration, tube formation, and autophagy under HG were explored to support novel therapeutic strategies by targeting YAP1.

## 2 Materials and methods

### 2.1 Cell culture and transfection

The RF/6A cell line (Catalog Number: CRL-1780), comprising rhesus choroid-retinal endothelial cells, was procured from the ATCC (ATCC, Manassas, VA, USA). The human primary retinal microvascular endothelial cells (HPRMVECs) are obtained from Cell Biologics (catalog number: H-6065, Cell Biologics, Chicago, IL, USA). For incubation, the cells were exposed to fetal bovine serum (FBS) (10%) (12103C, Sigma, Rockville, MD, USA) and penicillin (100 U/mL)–streptomycin (100 µg/mL) (catalog number: 15140122, Invitrogen, Carlsbad, CA, USA)-contained RPMI 1640 medium (catalog number: 11875093, Gibco, Grand Island, NY, USA) within an environment maintained at 5% CO_2_ and a stable temperature of 37°C.

In the transfection experiments, cells were cultured in six-well plates until they attained 90% confluence to ensure optimal transfection conditions. Transfection was performed using two distinct reagents to introduce small-interfering RNAs (siRNAs). Specifically, cells were transfected with either YAP1-targeting siRNA (si-YAP1) or a scrambled negative control siRNA (si-NC) procured from Gene Pharma (Shanghai, China). The transfection with RNAiMAX (Invitrogen) was conducted according to the manufacturer’s protocol to ensure high transfection efficiency with minimal cytotoxicity. Subsequently, a second transfection was carried out using Lipofectamine 2000 (Invitrogen), strictly following the detailed steps outlined in the product’s manual. This dual transfection approach was designed to maximize the silencing efficiency while validating the reproducibility of the gene silencing effect.

### 2.2 Establishment of DR mouse models

In the study, a total of 15 six-week-old C57BL/6J mice (Shanghai Model Organisms, Shanghai, China) were systematically divided into two distinct groups: one subjected to dietary restriction (DR, *n* = 10) and the other serving as the normal control (Ctr, *n* = 5). In the former group, dietary restriction was induced by administering a high-fat diet coupled with daily intraperitoneal injections of streptozocin (STZ) (S0130, Sigma) at a dosage of 40 mg/kg for a period of five consecutive days. Equivalent injections of vehicle buffer were administered to the normal control (Ctr) group. Systematic daily assessments of blood glucose levels were performed before and after the injection regimen. Mice presenting with random blood glucose concentrations greater than 16.7 mmol/L were categorized as diabetic. Throughout the study, the mice received intraperitoneal injections of 6 mg/kg Verteporfin (catalog number: S1786, Selleckchem, Houston, TX, USA) every other day, starting from the initiation of DR construction (*n* = 5). A comparable volume of phosphate-buffered saline (PBS) was utilized as a vehicle control (*n* = 5).


**Ethical approval:** The research related to animal use has been complied with all the relevant national regulations and institutional policies for the care and use of animals and has been approved by the ethics committee of Jinzhou Medical University (No.240027).

### 2.3 Isolation and culture of primary mouse retinal endothelial cells

Based on previously published procedures [10,11], the primary retinal endothelial cells were isolated from differentially treated mice. Briefly, mice were euthanized and their eyes removed and placed in 1× ice-cold PBS within a 48-well plate. Under a dissection microscope at 4.0× magnification, each eye was dissected by stabilizing the optic nerve with forceps, making a circular tear around the cornea, and peeling off the sclera to isolate the retina, which was then placed in microcentrifuge tubes containing ice-cold PBS. Retinas were digested in a warmed solution of DMEM with 10% FBS and 1 mg/mL Collagenase Type II (17101015, Gibco), pipetted every 5 min during a 20-min incubation at 37°C. Following digestion, the retinal tissues were processed into a single-cell suspension and stained with FITC-CD31 (160211, BioLegend, San Diego, CA, USA) and APC-CD45 (157605, BioLegend) antibodies, and the CD31^+^CD45^−^ endothelial cells were isolated via flow cytometry and cultured in DMEM supplemented with 10% FBS.

### 2.4 Cell vitality assay

The RF/6A cells and HPRMVECs were cultured under glucose at different concentrations (0, 5, 10, and 20 mM). For cell viability analysis, the cell counting kit-8 (CCK-8) (C0038, Beyotime, Shanghai, China) was employed. Briefly, the protocol entailed the seeding of cells at a density of 5,000 cells/well within 96-well plates for the experiment. After different treatments for 24 h, 10 μL of CCK‐8 solution was added to each well. Following the incubation of samples at 37°C for a duration of 4 h, optical density measurements at 450 nm were conducted using a Thermo Fisher Scientific microplate reader (Thermo Fisher Scientific, Waltham, MA, USA). The cells treated with 20 mM l-glucose (C5500, Sigma) were used as the group of osmotic control.

### 2.5 Quantitative real-time PCR

Utilizing the TRIzol reagent (catalog number: 15596026, Invitrogen), total RNA was isolated from the cellular samples. This RNA, in quantities of one microgram, was then converted into cDNA using the PrimeScript RT Reagent Kit (RR037B, Takara, Tokyo, Japan). The quantitative real-time PCR was carried out with SYBR Premix Ex (RR420A, Takara) on the ABI7500 quantitative PCR system (Thermo Fisher Scientific). The primers used are as follows: Monkey YAP1, forward 5′-TAGCCCTGCGTAGCCAGTTA-3′, reverse 5′-TCATGCTTAGTCCACTGTCTGT-3′; Monkey β-actin, forward 5′-CATGTACGTTGCTATCCAGGC-3′, reverse 5′-CTCCTTAATG TCACGCACGAT-3′. Human YAP1, forward 5′-TGTCCCAGATGAACGTCACAGC-3′, reverse 5′-TGGTGGCTGTTTCACTGGAGCA-3′; Human β-actin, forward 5′-CACCATTGGCAATGAGCGGTTC-3′, reverse 5′-AGGTCTTTGCGGATGTCCACGT-3′. The relative gene expression was calculated by the 2^−ΔΔCt^ method, with β-actin serving as a housekeeping gene.

### 2.6 Immunofluorescent staining

After washing using PBS, fixation in paraformaldehyde (4%), immersion of the cells onto the slide was conducted using 0.5% Triton X‐100 (X100, Sigma). Following this, the slides underwent an overnight incubation at 4°C with an anti-LC3 antibody (ab48394, 1:100 dilution; Abcam, Waltham, Boston, USA), and then with FITC-labeled immunoglobulin G (ab81051, Abcam) at a 1:100 dilution at 37°C. Post-incubation, the slides were treated with 4′,6-diamidino-2-phenylindole (DAPI) (ab285390) for 5 min in the dark, and subsequently, images were captured using a fluorescence microscope (IX81, Olympus, Greece) from Olympus.

### 2.7 Western blot

Following the extraction of total proteins from the cells, these proteins were then analyzed using Western blot methodology. Concisely, the separation of the protein samples was achieved using sodium dodecyl sulfate-polyacrylamide gels, followed by their transfer to polyvinylidene fluoride membranes (IPVH00010, Millipore, CA, USA). The membranes were subjected to blocking using 5% nonfat milk for a duration of 2 h, followed by overnight incubation at 4°C with the specified primary antibodies. Subsequently, the membranes underwent a 1-h incubation with horseradish peroxidase-conjugated secondary antibodies, followed by treatment with the ECL substrate (A38554, Thermo Fisher Scientific). Analysis of the protein bands was conducted using Quantity One software (Bio-Rad, Hercules, CA, USA).

### 2.8 Detection of cell apoptosis using flow cytometry

The determination of apoptotic rates involved treating the cells with Annexin V and PI, as contained in the Annexin V-FITC Apoptosis Detection Kit (556547, BD, Bangladesh). The treatment volume was 100 µL, and it was carried out on ice for 30 min in a light-free environment. The cells were subjected to three PBS washes, followed by the addition of 400 µL of binding buffer. Furthermore, the sample was examined utilizing a BD FACSCalibur flow cytometer to determine the apoptosis. Data analysis was conducted with BD CellQuest™ Pro software (BD, USA) to calculate the proportion of apoptotic cells within each group.

### 2.9 TUNEL assay

Cell fixation was accomplished by a 3-min immersion of the cells in paraformaldehyde (4%, pH 7.4) at −20°C. After PBS rinsing, cell permeabilization was achieved using 0.1% Triton X-100. Subsequently, the specimens underwent a PBS wash and were then exposed to a TUNEL reagent comprising fluorescent isothiocyanate dUTP and terminal deoxynucleotidyl transferase (ab66110, Abcam). Following the incubation period, the cells were subjected to staining with 1 μg/mL DAPI (ab285390, Abcam) for 30 min, facilitating the examination of the cell nucleus under UV light microscopic observation. Subsequently, all morphometric measurements were independently assessed by a minimum of three unbiased individuals in a blinded fashion.

### 2.10 Transwell chamber assay

In the first step, transwell inserts (3470, Corning, Corning, NY, USA) were utilized where 200 μL of cell suspensions, with a concentration of 5 × 10^5^ cells/mL, were placed in the top chamber. In parallel, the lower compartment was filled with 800 μL of a medium that contained 10% FBS. Subsequently, the cells underwent a 48-h incubation period at 37°C. Post incubation, the migrated cells on the bottom surface were subjected to fixation with 70% ethanol and staining with 0.5% crystal violet (V5265, Sigma). For the final analysis, the enumeration of cells that migrated was conducted in five randomly selected fields, observed through an Olympus microscope (Olympus, Tokyo, Japan).

### 2.11 Matrigel assay

In the tube formation assay, cells were subjected to overnight incubation at 37°C on plates pre-coated with 200 μL of Matrigel (354234, BD Biosciences, Bangladesh). Photographic documentation of the tube morphology was achieved using a microscope at 100× magnification. Subsequently, the quantification of branching points was carried out utilizing ImageJ software (NIH, Bethesda, MD).

### 2.12 Statistical analysis

Quantitative data in this study are expressed in terms of means ± standard deviation, with these measures being the outcome of at least three independent experimental procedures. For statistical evaluation, SPSS 19.0 (IBM, Armonk, NY) was utilized, applying a one-way analysis of variance and subsequent least significant difference post-hoc analysis. Values yielding a *P*-value of less than 0.05 were deemed to have reached statistical significance.

## 3 Results

### 3.1 HG activates YAP1 expression in RF/6A cells and HPRMVECs

**Figure 1:**
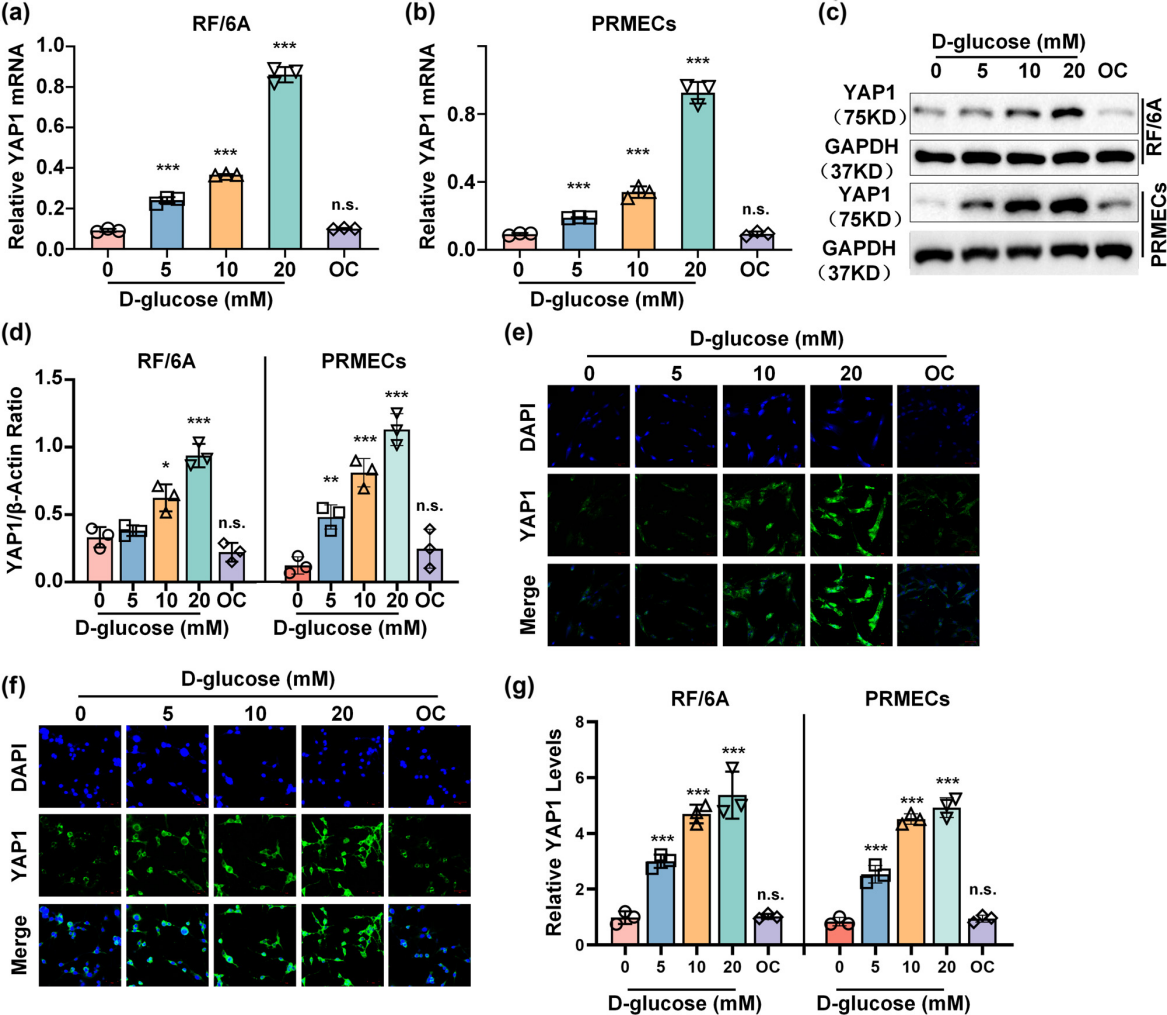
HG activates YAP1 expression in RF/6A cells and HPRMVECs. (a–d) The retinal endothelial cells (RF/6A cells) and human primary microvascular cells (HPRMVECs) were cultured under glucose at different concentrations (0, 5, 10, and 20 mM). The mRNA (a and b) and protein (c and d) expression levels of YAP1 in RF/6A cells and HPRMVECs were determined by using qRT-PCR and Western blot analysis, respectively. Representative images (e and f) and bar graph (g) representing the expression of YAP1 detected by immunofluorescent staining in RF/6A cells (e and g) and HPRMVECs (f and g) after indicated treatments. *P < 0.05, **P < 0.01, and ***P < 0.001.

First, we evaluated the expression of YAP1 in RF/6A cells and HPRMVECs under glucose at different concentrations (0, 5, 10, and 20 mM). Consequently, quantitative reverse transcription polymerase chain reaction (qRT-PCR) and Western blot analysis revealed that the mRNA (Figure 1a and b) and protein (Figure 1c and d) expression levels of YAP1 in RF/6A cells (Figure 1a, c, and d) and HPRMVECs (Figure 1b–d) significantly elevated under glucose treatment in a dose-dependent manner (Figure 1a–d). Immunofluorescent staining also demonstrated the activation of YAP1 in RF/6A cells and HPRMVECs exposed to glucose administration (Figure 1e and f). Collectively, these findings suggested that YAP1 was activated in retinal endothelial cells exposed to glucose.

### 3.2 Inhibition of YAP1 suppresses viability and decreases apoptosis of retinal endothelial cells under HG

**Figure 2:**
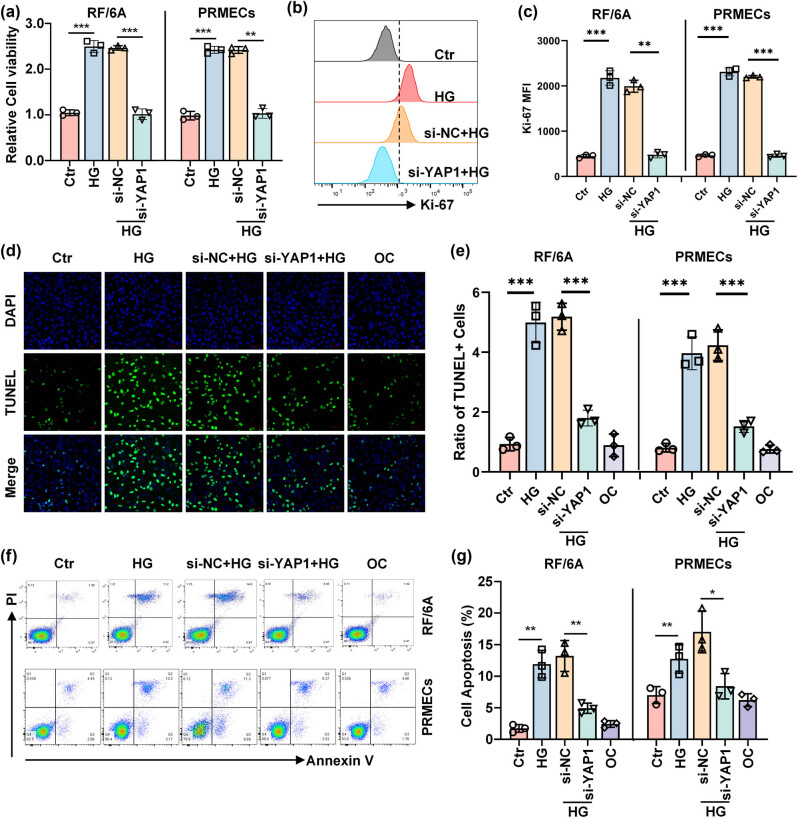
Inhibition of YAP1 elevates viability and decreases apoptosis caused by HG. (a) Representative bar graph depicts the viability of RF/6A cells and HPRMVECs detected by CCK-8 assay after indicated treatments. Representative histograms (b) and bar graph (c) represent the expression of Ki-67 detected by flow cytometry in RF/6A cells and HPRMVECs. Representative images (d) and bar graph (e) showing the apoptosis of RF/6A cells and HPRMVECs detected by TUNEL staining. Representative pseudocolor plots (f) and bar graph (g) representing the apoptosis of RF/6A cells and HPRMVECs monitored by Annexin V/PI staining. *P < 0.05, **P < 0.01, and ***P < 0.001.

Given the glucose-induced activation of YAP1, we next silenced the expression of YAP and then evaluated the functional relevance of YAP1 inhibition in glucose-treated RF/6A cells and HPRMVECs. Western blot analysis revealed that, compared to cells transfected with non-targeting control siRNA (si-NC), cells transfected with YAP1-specific siRNA (si-YAP1) demonstrated a significant reduction in YAP1 protein levels, confirming the effective silencing of YAP1 in RF/6A cells (Figure S1a and b) and HPRMVECs (Figure S1a and b). Subsequent CCK‐8 assay indicated that HG at 20 mM significantly promoted the viability of RF/6A cells and HPRMVECs (Figure 2a). However, inhibition of YAP1 partially reversed such effects (Figure 2a). This cell viability result was also supported by the expression of Ki-67 detected using the flow cytometry method. We observed that compared to the non-treated control cells, the cells treated with HG exhibited significantly elevated Ki-67 expression; however, this HG exposure-caused elevation of Ki-67 expression was dramatically restored by the inhibition of YAP1 (Figure 2b and c), suggesting the protection of YAP1 inhibition on HG-induced cell injury. Moreover, under the HG condition, TUNEL assay (Figure 2d and e) and flow cytometry (Figure 2f and g) analysis demonstrated an increased apoptosis rate of RF/6A cells and HPRMVECs (Figure 2d–g). Nevertheless, cell apoptosis significantly decreased following the knockdown of YAP1 even under HG treatment (Figure 2d–g). Collectively, these data revealed that suppression of YAP1 could be protective against glucose-induced retinal endothelial cell injury.

### 3.3 Inhibition of YAP1 suppresses migration and tube formation in retinal endothelial cells under HG

**Figure 3:**
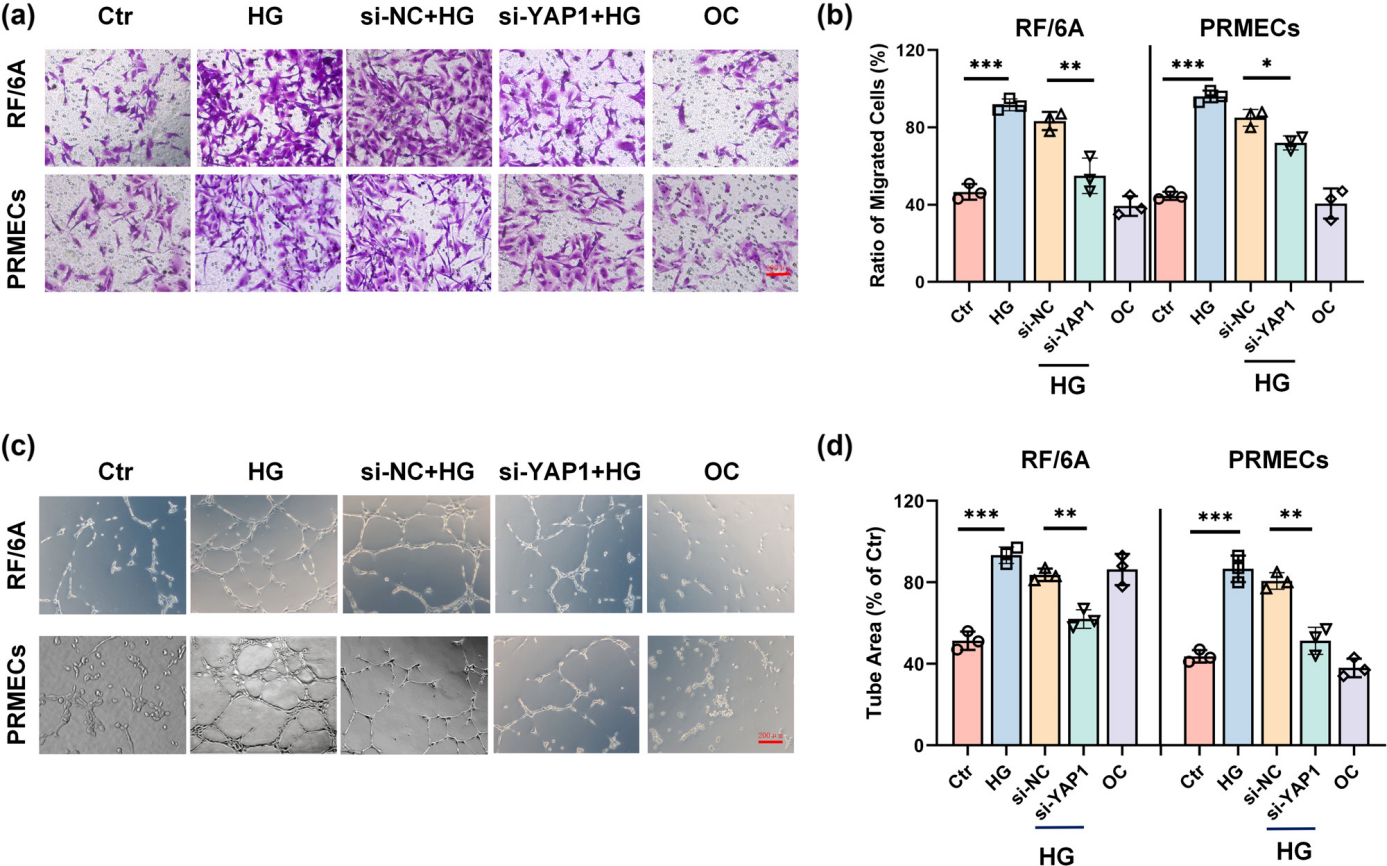
Inhibition of YAP1 enhances migration and tube formation under HG exposure. Representative images (a) and bar graph (b) show the migration of RF/6A cells and HPRMVECs after different treatments. Representative images (c) and bar graph (d) depicting the tube formation of RF/6A cells and HPRMVECs after indicated treatments. *P < 0.05, **P < 0.01, and ***P < 0.001.

Transwell assay indicated that HG treatment significantly increased RF/6A cell and HPRMVEC migration compared with those cells in the control group, and inhibition of YAP1 obviously reversed such effects (Figure 3a and b). In addition, the Matrigel assay demonstrated that knockdown of YAP1 dramatically inhibited the number of tube formation of RF/6A cells and HPRMVECs under HG treatment (Figure 3c and d).

### 3.4 Inhibition of YAP1 suppresses autophagy caused by HG

**Figure 4:**
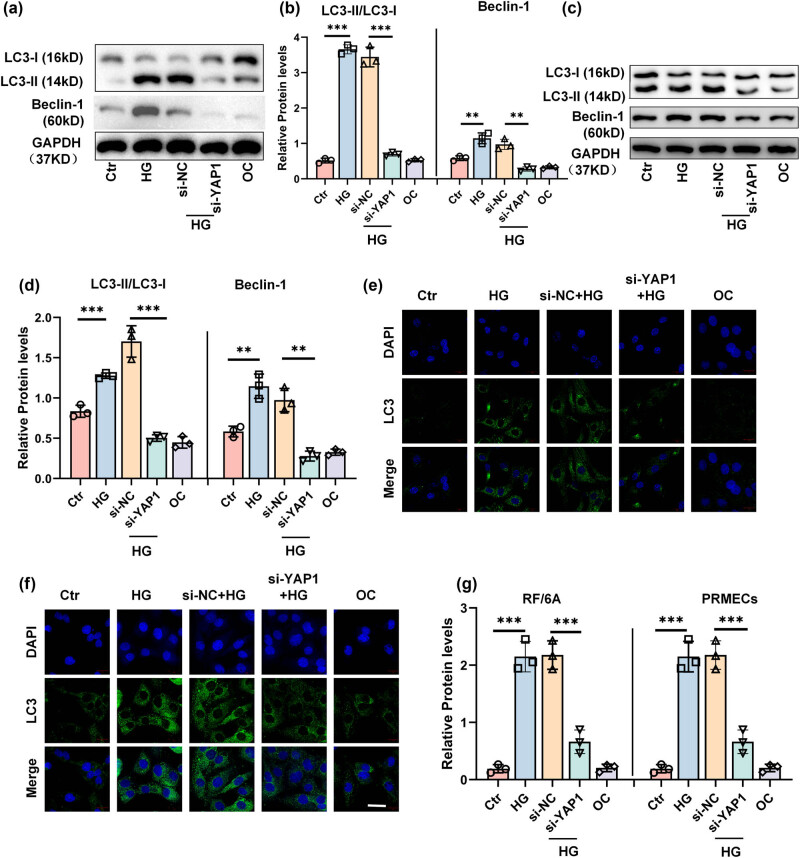
Inhibition of YAP1 suppresses autophagy caused by HG. Representative blots (a and c) and bar graph (b and d) depicting the expression of LC3 and Beclin-1 in RF/6A cells (a and b) and HPRMVECs (c and d) detected by Western blot after indicated treatments. Representative images (e and f) and bar graph (g) representing the GFP-LC3 puncta accumulation in RF/6A cells (e and g) and HPRMVECs (f and g) measured by immunofluorescent staining after indicated treatments. **P < 0.01 and ***P < 0.001.

Accumulated studies have documented that LC3 is a key protein in the autophagy pathway, often associated with autophagosome formation, a process that can be linked to both survival and cell death mechanisms [12,13,14,15]. Beclin-1, another crucial autophagy-related protein, plays a pivotal role in the initiation phase of autophagy and has been implicated in the regulation of apoptosis [16,17,18,19]. Its interaction with other autophagic and apoptotic regulators can influence cell fate decisions under stress conditions. Therefore, we evaluated the effects of YAP1 inhibition on the autophagy of RF/6A cells and HPRMVECs in the presence of HG. Western blot analysis showed that LC3 II/I ratio (Figure 4a–d) and Beclin‐1 (Figure 4a–d) expressions significantly elevated in HG-treated RF/6A cells (Figure 4a and b) and HPRMVECs (Figure 4c and d). In addition, suppression of YAP1 reduced LC3 II/I ratio and Beclin‐1 expression in RF/6A cells (Figure 4a and b) and HPRMVEC (Figure 4c and d) in the presence of HG (Figure 4a). Consistently, we also observed significantly increased accumulation of GFP-LC3 puncta after HG treatment in RF/6A cells (Figure 4e and g) and HPRMVECs (Figure 4f and g), whereas such effects were obviously alleviated by the suppression of YAP1 (Figure 4e–g). These results showed that suppression of YAP1 alleviated HG-induced autophagy in RF/6A cells and HPRMVECs.

### 3.5 Inhibition of YAP1 blocked the nucleus translation of NF-κB caused by HG exposure

**Figure 5:**
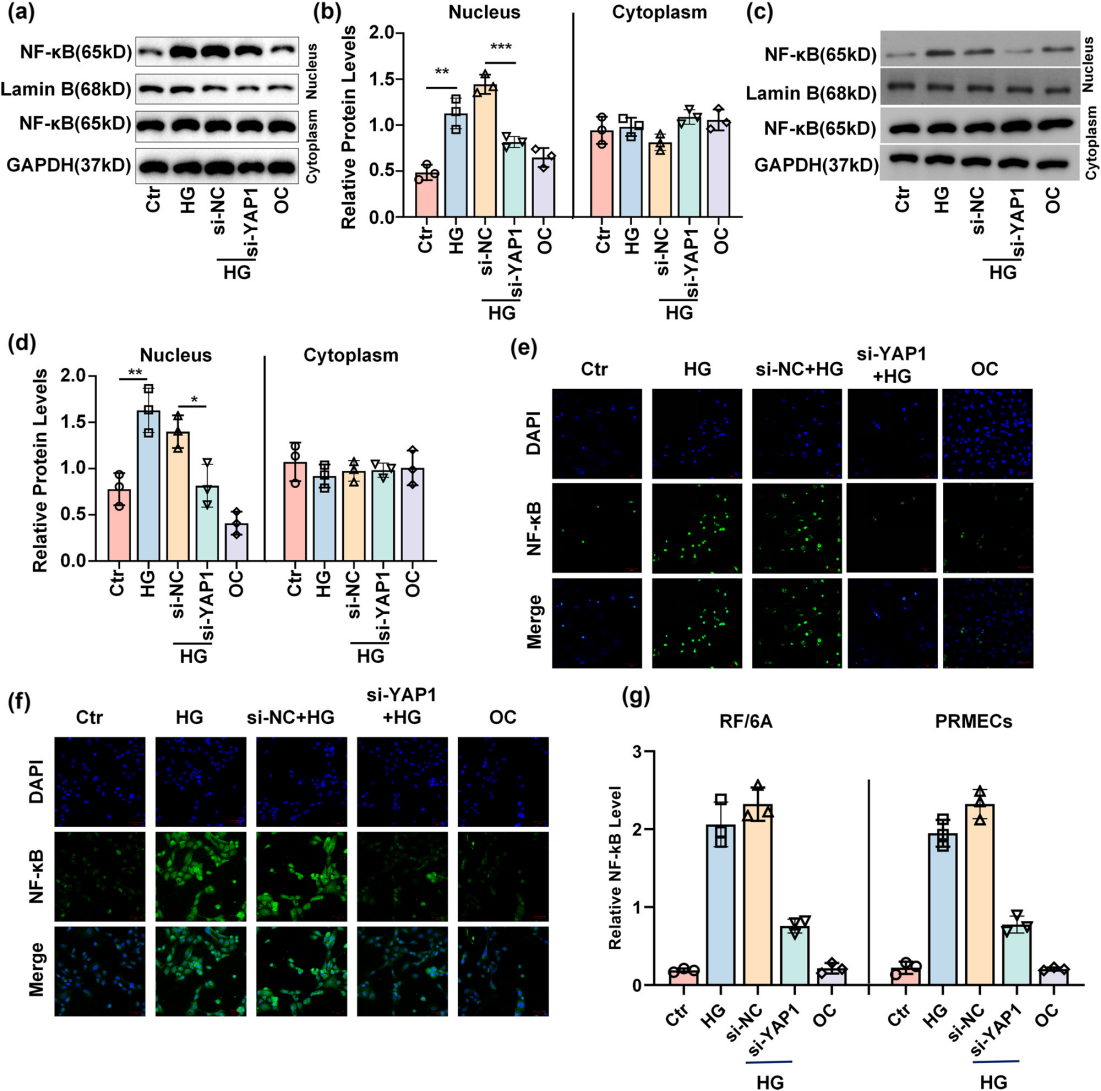
Inhibition of YAP1 blocks the nucleus translocation of NF-κB induced by HG exposure. Representative blots (a and c) and bar graph (b and d) showing the accumulation of NF-κB in the nucleus and cytoplasm of RF/6A cells (a and b) and HPRMVECs (c and d) detected by Western blot after indicated treatments. Representative images (e and f) and bar graph (g) representing the translation of NF-κB in the nucleus and cytoplasm of RF/6A cells (e and g) and HPRMVECs (f and g) detected by immunofluorescent staining after indicated treatments. *P < 0.05, **P < 0.01, and ***P < 0.001.

NF-κB is a key transcription factor involved in inflammatory responses, immune system activation, and cell survival pathways [20,21,22,23]. Therefore, we further evaluated the effects of NF-κB signaling in RF/6A cells and HPRMVECs under HG. Western blot analysis showed that after HG exposure, both of the RF/6A cells (Figure 5a and b) and HPRMVECs (Figure 5c and d) exhibited significantly elevated accumulation of NF-κB in the nucleus, but no obvious changes of the cytoplasmic NF-κB were observed. In addition, suppression of YAP1 dramatically decreased the translocation of NF-κB in the nucleus of RF/6A cells (Figure 5a and b) and HPRMVECs (Figure 5c and d) exposed to HG (Figure 5a–d). Moreover, immunofluorescence staining analysis demonstrated that HG increased the nucleus accumulation of NF-κB in RF/6A cells (Figure 5e and g) and HPRMVECs (Figure 5f and g), whereas suppression of YAP1 blocked its translocation (Figure 5e–g). These findings suggested that YAP1 suppression blocked HG-induced nucleus translocation of NF-κB in retinal endothelial cells.

### 3.6 Inhibition of YAP1 protected the retinal endothelial cells from HG-caused injury *in vivo*

**Figure 6:**
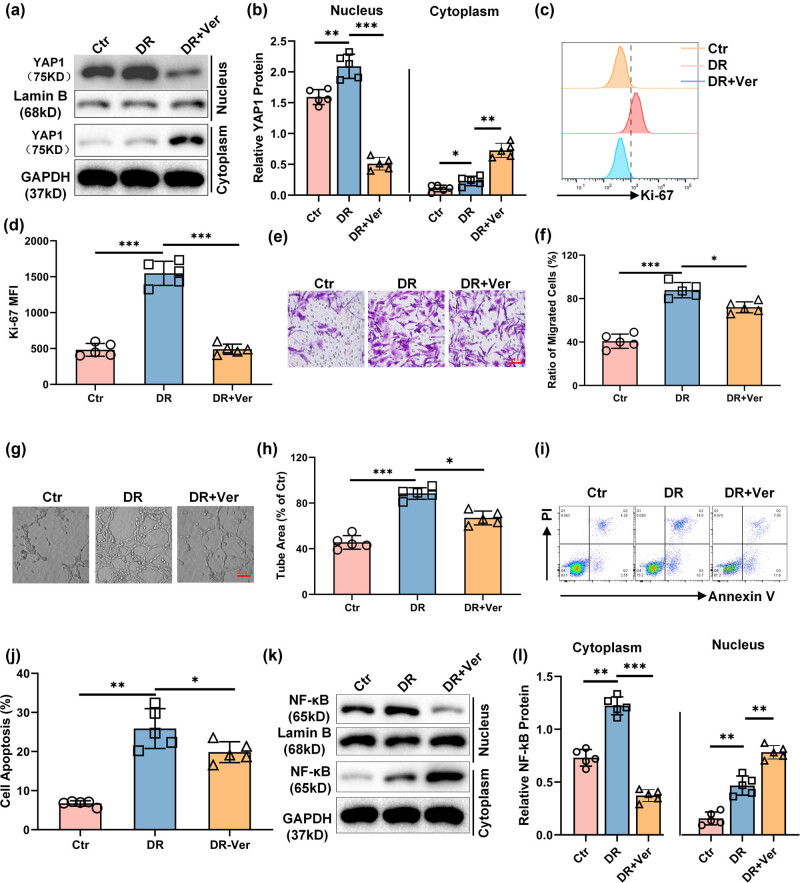
Inhibition of YAP1 protected the retinal endothelial cells from HG-caused injury *in vivo.* Representative blot (a) and bar graph (b) depicting the expression of YAP1 in the mice retinal microvascular epithelial cells isolated from differently treated DR mice. Representative histograms (c) and bar graph (d) showing the expression Ki-67 detected using flow cytometry in the retinal microvascular epithelial cells isolated from differently treated DR mice. Representative images (e) and bar graph (f) showing the migration of the retinal microvascular epithelial cells isolated from differently treated DR mice. Representative images (g) and bar graph (h) representing the tube formation of the retinal microvascular epithelial cells isolated from differently treated DR mice. Representative pseudocolor plots (i) and bar graph (j) depicting the apoptosis of the retinal microvascular epithelial cells isolated from differently treated DR mice. Representative blot (k) and bar graph (l) representing the accumulation of NF-κB in the nucleus and cytoplasm of the retinal microvascular epithelial cells isolated from differently treated DR mice. *P < 0.05, **P < 0.01, and ***P < 0.001.

Verteporfin is predominantly known for its application in photodynamic therapy, particularly for the treatment of neovascular macular degeneration. In recent studies, Verteporfin has also been shown to inhibit YAP1-TEAD complex formation, thereby blocking the YAP1 signaling pathway, which is crucial in regulating cellular proliferation and apoptosis in various types of cells [24,25,26,27,28]. Thus, to further verify the protective effect of YAP1 inhibition on HG-caused retinal endothelial cell injury *in vivo*, an STZ-induced DR mouse model was employed and Verteporfin was injected to suppress the activation of YAP1 *in vivo*. Then, the mouse retinal endothelial cells were isolated, and the injury of the retinal endothelial cells caused by DR was monitored. We observed that, in comparison to the cells isolated from the mice of Ctr group, the cells from the DR mice exhibited significantly elevated YAP1 expression (Figure 6a and b), increased cell growth (Figure 6c and d), enhanced cell migration (Figure 6e and f), promoted tube formation (Figure 6g and h), augmented cell apoptosis (Figure 6i and j), and increased NF-κB accumulation in nucleus (Figure 6k and l), which were consistent with our above observations *in vitro*. However, obvious improvement of these HG-induced cell injuries was observed in the DR mice administrated with Verteporfin, suggesting the protection of YAP1 inhibition on the DR-caused retinal epithelial cell injury *in vivo*.

## 4 Discussion

DR is a common diabetes‐associated microvascular complication. Controlling HG-induced angiogenesis is critical to prevent the development of DR. Therefore, understanding the mechanisms by which HG promotes angiogenesis is essential to develop novel therapeutic targets for the treatment of DR [29,30]. In the present study, our data demonstrated that HG exposure could activate YAP1 expression, while inhibition of YAP1 exhibits a protective role against HG-induced RVEC injury, representing a novel target for the treatment of DR.

Earlier investigations have revealed the integral role of YAP1 in the realms of organ development, tissue repair, and retinal angiogenesis [31]. We found that YAP1 activation increases with higher glucose levels, suggesting its role in triggering endothelial dysfunction in diabetes. Supporting studies have observed similar increases in YAP protein in the cells of diabetic mice, indicating a consistent response to HG levels. Furthermore, reducing YAP1 activity in our experiments led to lower cell proliferation in HG conditions. This suggests that targeting YAP1 could help control excessive cell growth seen in diabetic vascular complications, pointing to its potential as a therapeutic target [32]. YAP1 has also been identified as a necessary factor for angiogenesis in retinal microvascular endothelial cells (RMECs) when responding to HG conditions [33,34]. In our study, suppression of YAP1 was found to increase cell viability and suppress apoptosis in HG-treated retinal endothelial cells. Moreover, inhibition of YAP1 could promote tube formation and migration of retinal endothelial cells in response to HG, suggesting the protective role of knocking YAP1 expression in retinal endothelial cells.

Autophagy is a kind of orchestrated lysosomal degradation process in response to external or internal stimuli, to maintain cellular homeostasis [35]. Previously, murine RMECs exhibited increased Beclin-1, Atg3, and Atg5 expressions and LC3BB-II/I ratio under hypoxia conditions. The underlying mechanism involved the activation of the AMPK/mTOR signaling pathway [36]. In addition, activation of autophagy has been reported in HG-treated umbilical vein endothelial cells *in vitro* and diabetic retinal vasculature *in vivo*, respectively [37]. A recent study has suggested that angiogenic factor with G patch and FHA domains 1 (AGGF1) impaired autophagy flux and LC3 and Beclin-1 expressions in response to hyperoxia, thereby alleviating RVEC injury [38]. In the present study, LC3 II/I ratio and Beclin‐1 expression in retinal endothelial cells were significantly elevated after HG treatment. Conversely, suppression of YAP1 decreased LC3 II/I ratio and Beclin‐1 expression as well as GFP-LC3 puncta accumulation, suggesting that silencing YAP1 expression protects against HG-induced retinal endothelial cell injury by suppressing autophagy.

Abnormal expression and activation of the transcription factor NF-κB, a central regulator of cell survival, have been linked to the development of multiple human diseases [39]. NF-κB nucleus translocation as well as the enhanced downstream proinflammatory response have been reported to be elevated in response to HG [40]. Furthermore, autophagic flux in human intestinal epithelial cells is regulated by the NF-κB pathway, suggesting the close relationship between autophagy and NF-κB [41]. In the present study, HG-induced dramatic translocation of nuclear NF-κB was blocked by suppression of YAP1 in retinal endothelial cells.

Additionally, in our study, an unexpected similarity in the effects of si-NC and si-YAP1 on tube formation was observed (Figure 3c). This phenomenon prompts an examination of potential underlying mechanisms and the specificity of the siRNA used in these experiments. One plausible explanation for this observation is the potential off-target effects associated with the non-targeting control siRNA. Such effects are a known challenge in RNA interference studies and can result from partial complementarity of the siRNA sequence to non-target transcripts, inadvertently leading to unintended gene silencing. Another consideration is the possibility of redundancy and compensation within the signaling pathways that regulate tube formation. The similar outcomes observed with both si-NC and si-YAP1 suggest that other signaling molecules may compensate for the loss of YAP1, thus sustaining angiogenic capabilities under these experimental conditions. Further investigations into the molecular networks associated with YAP1 could uncover these compensatory mechanisms and provide deeper insights into the cellular pathways influencing angiogenesis.

In summary, our study presents compelling evidence that YAP1 inhibition confers protection against HG-induced injury in retinal endothelial cells, a key pathological feature of DR. The modulation of YAP1 not only improves cell viability and reduces apoptosis but also enhances endothelial cell migration and tube formation, crucial for maintaining vascular integrity under diabetic conditions. These findings underscore the potential of YAP1 as a novel therapeutic target for mitigating retinal endothelial dysfunction associated with diabetes. Additionally, the mechanistic insights from our study indicate that YAP1 may act as a regulator of NF-κB nuclear translocation, a process integral to inflammatory responses in endothelial cells. By inhibiting YAP1, we observed a reversal of glucose-induced NF-κB activity, suggesting a link between YAP1 signaling and inflammatory pathways in the diabetic milieu. This relationship provides a foundational basis for further exploration into how YAP1-mediated signaling pathways could be manipulated to prevent or delay the onset of DR.

Future research should focus on validating these findings in longer-term diabetic models and in human subjects to understand the chronic effects of YAP1 inhibition. It would also be valuable to explore the interaction between YAP1 and other molecular pathways implicated in diabetic complications, such as oxidative stress and advanced glycation end-product signaling. Understanding these interactions could open up new avenues for comprehensive therapeutic strategies that target multiple aspects of DR pathogenesis. Clinically, targeting YAP1 offers a promising approach for early intervention in DR. As DR remains a major cause of vision loss in diabetic patients, early therapeutic strategies that can prevent or slow the progression of endothelial dysfunction could significantly improve patient outcomes. Developing YAP1 inhibitors or modulators as part of a treatment regimen for diabetes could potentially reduce the incidence or severity of DR, offering a substantial benefit over current therapies that mainly focus on the advanced stages of the disease.

## Supplementary Material

Supplementary Figure

## References

[1] Glovaci D, Fan W, Wong ND. Epidemiology of diabetes mellitus and cardiovascular disease. Curr Cardiol Rep. 2019;21(4):21. 10.1007/s11886-019-1107-y 30828746

[2] Wang W, Lo ACY. Diabetic retinopathy: pathophysiology and treatments. Int J Mol Sci. 2018;19(6):1816–38. 10.3390/ijms19061816 PMC6032159 29925789

[3] Whitehead M, Wickremasinghe S, Osborne A, Van Wijngaarden P, Martin KR. Diabetic retinopathy: a complex pathophysiology requiring novel therapeutic strategies. Expert Opin Biol Ther. 2018;18(12):1257–70. 10.1080/14712598.2018.1545836 PMC6299358 30408422

[4] Levine B, Kroemer G. Biological functions of autophagy genes: a disease perspective. Cell. 2019;176(1–2):11–42. 10.1016/j.cell.2018.09.048 PMC6347410 30633901

[5] Gong Q, Wang H, Yu P, Qian T, Xu X. Protective or harmful: the dual roles of autophagy in diabetic retinopathy. Front Med (Lausanne). 2021;8:644121. 10.3389/fmed.2021.644121 PMC8026897 33842506

[6] Mathew B, Chennakesavalu M, Sharma M, Torres LA, Stelman CR, Tran S, et al. Autophagy and post-ischemic conditioning in retinal ischemia. Autophagy. 2021;17(6):1479–99. 10.1080/15548627.2020.1767371 PMC8205079 32452260

[7] Liang N, Zhang C, Dill P, Panasyuk G, Pion D, Koka V, et al. Regulation of YAP by mTOR and autophagy reveals a therapeutic target of tuberous sclerosis complex. J Exp Med. 2014;211(11):2249–63. 10.1084/jem.20140341 PMC4203941 25288394

[8] Yu Y, Su X, Qin Q, Hou Y, Zhang X, Zhang H, et al. Yes-associated protein and transcriptional coactivator with PDZ-binding motif as new targets in cardiovascular diseases. Pharmacol Res. 2020;159:105009. 10.1016/j.phrs.2020.105009 32553712

[9] He J, Bao Q, Yan M, Liang J, Zhu Y, Wang C, et al. The role of Hippo/yes-associated protein signalling in vascular remodelling associated with cardiovascular disease. Br J Pharmacol. 2018;175(8):1354–61. 10.1111/bph.13806 PMC5866970 28369744

[10] Chavkin NW, Cain S, Walsh K, Hirschi KK. Isolation of murine retinal endothelial cells for next-generation sequencing. J Vis Exp. 2021;(176):10.3791/63133. 10.3791/63133 PMC8641104 34694293

[11] Su X, Sorenson CM, Sheibani N. Isolation and characterization of murine retinal endothelial cells. Mol Vis. 2003;9:171–8. 12740568

[12] Priem D, Huyghe J, Bertrand MJ. LC3-independent autophagy is vital to prevent TNF cytotoxicity. Autophagy. 2023;19(9):2585–9. 10.1080/15548627.2023.2197760 PMC10392734 37014272

[13] Parzych KR, Klionsky DJ. An overview of autophagy: morphology, mechanism, and regulation. Antioxid Redox Signal. 2014;20(3):460–73. 10.1089/ars.2013.5371 PMC3894687 23725295

[14] Huang R, Liu W. Identifying an essential role of nuclear LC3 for autophagy. Autophagy. 2015;11(5):852–3. 10.1080/15548627.2015.1038016 PMC4509442 25945743

[15] Tanida I, Ueno T, Kominami E. LC3 and autophagy. Methods Mol Biol. 2008;445:77–88. 10.1007/978-1-59745-157-4_4 18425443

[16] Tran S, Fairlie WD, Lee EF. BECLIN1: protein structure, function and regulation. Cells. 2021;10(6):1522–41. 10.3390/cells10061522 PMC8235419 34204202

[17] Vega-Rubin-de-Celis S. The role of beclin 1-dependent autophagy in cancer. Biology (Basel). 2019;9(1):4–15. 10.3390/biology9010004 PMC7168252 31877888

[18] Kang R, Zeh HJ, Lotze MT, Tang D. The beclin 1 network regulates autophagy and apoptosis. Cell Death Differ. 2011;18(4):571–80. 10.1038/cdd.2010.191 PMC3131912 21311563

[19] Vega-Rubin-de-Celis S, Kinch L, Pena-Llopis S. Regulation of beclin 1-mediated autophagy by oncogenic tyrosine kinases. Int J Mol Sci. 2020;21(23):9210–30. 10.3390/ijms21239210 PMC7729755 33287140

[20] Liu T, Zhang L, Joo D, Sun SC. NF-kappaB signaling in inflammation. Signal Transduct Target Ther. 2017;2:17023. 10.1038/sigtrans.2017.23 PMC5661633 29158945

[21] Liu D, Zhong Z, Karin M. NF-kappaB: a double-edged sword controlling inflammation. Biomedicines. 2022;10(6):1250–65. 10.3390/biomedicines10061250 PMC9219609 35740272

[22] Roberti A, Chaffey LE, Greaves DR. NF-kappaB signaling and inflammation-drug repurposing to treat inflammatory disorders? Biology (Basel). 2022;11(3):372–90. 10.3390/biology11030372 PMC8945680 35336746

[23] Barnabei L, Laplantine E, Mbongo W, Rieux-Laucat F, Weil R. NF-kappaB: at the borders of autoimmunity and inflammation. Front Immunol. 2021;12:716469. 10.3389/fimmu.2021.716469 PMC8381650 34434197

[24] Giraud J, Molina-Castro S, Seeneevassen L, Sifre E, Izotte J, Tiffon C, et al. Verteporfin targeting YAP1/TAZ-TEAD transcriptional activity inhibits the tumorigenic properties of gastric cancer stem cells. Int J Cancer. 2020;146(8):2255–67. 10.1002/ijc.32667 31489619

[25] Wei L, Ma X, Hou Y, Zhao T, Sun R, Qiu C, et al. Verteporfin reverses progestin resistance through YAP/TAZ-PI3K-Akt pathway in endometrial carcinoma. Cell Death Discov. 2023;9(1):30. 10.1038/s41420-023-01319-y PMC9873621 36693834

[26] Wang C, Zhu X, Feng W, Yu Y, Jeong K, Guo W, et al. Verteporfin inhibits YAP function through up-regulating 14-3-3sigma sequestering YAP in the cytoplasm. Am J Cancer Res. 2016;6(1):27–37. PMC4759394 27073720

[27] Wei C, Li X. Verteporfin inhibits cell proliferation and induces apoptosis in different subtypes of breast cancer cell lines without light activation. BMC Cancer. 2020;20(1):1042. 10.1186/s12885-020-07555-0 PMC7599100 33121449

[28] Huang Y, Ahmad US, Rehman A, Uttagomol J, Wan H. YAP Inhibition by verteporfin causes downregulation of desmosomal genes and proteins leading to the disintegration of intercellular junctions. Life (Basel). 2022;12(6):792–808. 10.3390/life12060792 PMC9225343 35743822

[29] Hammes HP. Diabetic retinopathy: hyperglycaemia, oxidative stress and beyond. Diabetologia. 2018;61(1):29–38. 10.1007/s00125-017-4435-8 28942458

[30] Feldman-Billard S, Larger E, Massin P. Standards for screeningand surveillance of ocular complications in people with diabetes SFDsg. Early worsening of diabetic retinopathy after rapid improvement of blood glucose control in patients with diabetes. Diabetes Metab. 2018;44(1):4–14. 10.1016/j.diabet.2017.10.014 29217386

[31] Moya IM, Halder G. Hippo-YAP/TAZ signalling in organ regeneration and regenerative medicine. Nat Rev Mol Cell Biol. 2019;20(4):211–26. 10.1038/s41580-018-0086-y 30546055

[32] Chen J, Harris RC. Interaction of the EGF receptor and the hippo pathway in the diabetic kidney. J Am Soc Nephrol. 2016;27(6):1689–700. 10.1681/ASN.2015040415 PMC4884112 26453611

[33] Han N, Tian W, Yu N, Yu L. YAP1 is required for the angiogenesis in retinal microvascular endothelial cells via the inhibition of MALAT1-mediated miR-200b-3p in high glucose-induced diabetic retinopathy. J Cell Physiol. 2020;235(2):1309–20. 10.1002/jcp.29047 31313295

[34] Ortillon J, Le Bail JC, Villard E, Leger B, Poirier B, Girardot C, et al. High glucose activates YAP Signaling to promote vascular inflammation. Front Physiol. 2021;12:665994. 10.3389/fphys.2021.665994 PMC8213390 34149446

[35] Tangvarasittichai O, Tangvarasittichai S. Oxidative stress, ocular disease and diabetes retinopathy. Curr Pharm Des. 2018;24(40):4726–41. 10.2174/1381612825666190115121531 30644339

[36] Li R, Wang LZ, Du JH, Zhao L, Yao Y. Autophagy activation and the mechanism of retinal microvascular endothelial cells in hypoxia. Int J Ophthalmol. 2018;11(4):567–74. 10.18240/ijo.2018.04.05 PMC5902358 29675372

[37] Niu C, Chen Z, Kim KT, Sun J, Xue M, Chen G, et al. Metformin alleviates hyperglycemia-induced endothelial impairment by downregulating autophagy via the Hedgehog pathway. Autophagy. 2019;15(5):843–70. 10.1080/15548627.2019.1569913 PMC6526809 30653446

[38] Yao G, Li R, Du J, Yao Y. Angiogenic factor with G patch and FHA domains 1 protects retinal vascular endothelial cells under hyperoxia by inhibiting autophagy. J Biochem Mol Toxicol. 2020;34(11):e22572. 10.1002/jbt.22572 32633013

[39] Verzella D, Pescatore A, Capece D, Vecchiotti D, Ursini MV, Franzoso G, et al. Life, death, and autophagy in cancer: NF-kappaB turns up everywhere. Cell Death Dis. 2020;11(3):210. 10.1038/s41419-020-2399-y PMC7105474 32231206

[40] Liu H, Yu S, Xu W, Xu J. Enhancement of 26S proteasome functionality connects oxidative stress and vascular endothelial inflammatory response in diabetes mellitus. Arterioscler Thromb Vasc Biol. 2012;32(9):2131–40. 10.1161/ATVBAHA.112.253385 PMC3432586 22772755

[41] Zhou M, Xu W, Wang J, Yan J, Shi Y, Zhang C, et al. Boosting mTOR-dependent autophagy via upstream TLR4-MyD88-MAPK signalling and downstream NF-kappaB pathway quenches intestinal inflammation and oxidative stress injury. EBioMedicine. 2018;35:345–60. 10.1016/j.ebiom.2018.08.035 PMC6161481 30170968

