# Supplementary material for: YAP1 inhibition protects retinal vascular endothelial cells under high glucose by inhibiting autophagy
Source: Open Life Sci. 2025 Dec 24;20(1):20220970. doi: 10.1515/biol-2022-0970 (PMC12728944; doi:10.1515/biol-2022-0970)
Supplement: Supplementary Figure [file biol-2022-0970-sm.pdf]

Supplementary material

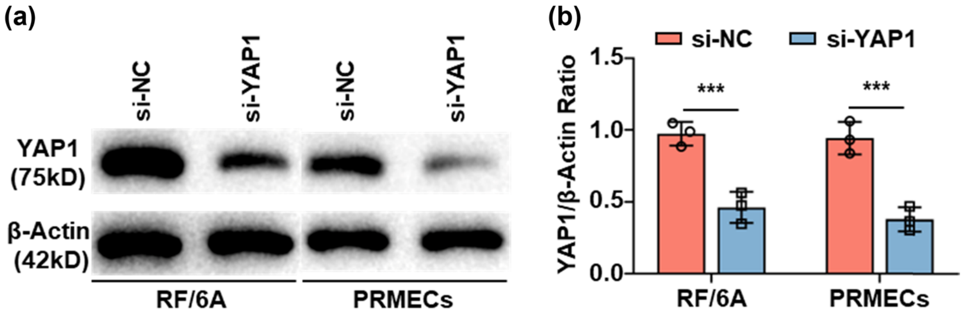

Figure S1: The protein levels of YAP1 in the RF/6A and PRMECs after indicated transfections were monitored by Western Blot method. Representative blots (a) and bar graph (b) represent the expression of YAP1 in differentially treated cells. ***, $P < 0.001$.
